# Letter to the Editor – Optimizing Culotte Technique with Double Kissing Balloon Inflation: A Response to Berkay Serter´s Letter to the Editor

**DOI:** 10.1007/s12265-025-10682-3

**Published:** 2025-08-18

**Authors:** Faridun Rahimi, Christoph Olivier, Jan Minners, Miroslaw Ferenc, Franz-Josef Neumann, Dirk Westermann

**Affiliations:** Department of Cardiology and Angiology, Medical Center - University of Freiburg, Faculty of Medicine, University of Freiburg, Suedring 15, 79189 Bad Krozingen, Germany

To the Editor,

We would like to thank the authors for their thoughtful comments regarding our article “‘Randomised comparison of culotte- versus double kissing crush stenting in de novo non-left main coronary bifurcation lesions: Rationale and design of the Bifurcation Bad Krozingen trial-3 (BBK-3)“ [1]. We appreciate the detailed discussion on double-kissing culotte (DK-culotte) and recognize that multiple approaches have been developed for managing coronary bifurcation lesions, including various bifurcation stenting techniques, which have been modified over the time [2]. The modification of the culotte technique to DK-culotte by an additional kissing manoeuvre after first stent implantation may help to optimise the apposition of the stent struts, particularly at the polygon of influence. We agree with the authors that there is a lack of substantiated data demonstrating an advantage for DK-culotte compared with culotte with regard to clinical endpoints. In a bench test DK-culotte was compared with culotte and DK-crush [3]. The results confirmed a significantly lower malapposition with DK-culotte compared with DK-crush. No statistical difference between DK-culotte and culotte was observed.

When the study design of BBK-3 was developed, the „classical “ culotte stenting was an established and well-studied 2 stent strategy for the treatment of bifurcation lesions. DK-culotte was developed after the start of the trial. To ensure consistency and reproducibility, culotte was retained for BBK-3, especially in the view of lack of well-founded randomized clinical data for DK-culotte.

The trial will provide meaningful information comparing the established bifurcation techniques culotte with DK-crush in non-left-main stenosis. Future studies may incorporate more recent stenting techniques such as DK-culotte and other techniques.

## Data Availability

No new data were generated or analyzed in support of this research.
